# Syndromes associated with frontotemporal lobar degeneration change response patterns on visual analogue scales

**DOI:** 10.1038/s41598-023-35758-5

**Published:** 2023-06-02

**Authors:** Rebecca S. Williams, Natalie E. Adams, Laura E. Hughes, Matthew A. Rouse, Alexander G. Murley, Michelle Naessens, Duncan Street, Negin Holland, James B. Rowe

**Affiliations:** 1 MRC Cognition and Brain Sciences Unit, University of Cambridge, Cambridge, UK; 2 Department of Clinical Neurosciences and Cambridge University Hospitals NHS Trust, University of Cambridge, Cambridge, UK

**Keywords:** Human behaviour, Neurological disorders

## Abstract

Self-report scales are widely used in cognitive neuroscience and psychology. However, they rest on the central assumption that respondents engage meaningfully. We hypothesise that this assumption does not hold for many patients, especially those with syndromes associated with frontotemporal lobar degeneration. In this study we investigated differences in response patterns on a visual analogue scale between people with frontotemporal degeneration and controls. We found that people with syndromes associated with frontotemporal lobar degeneration respond with more invariance and less internal consistency than controls, with Bayes Factors = 15.2 and 14.5 respectively indicating strong evidence for a group difference. There was also evidence that patient responses feature lower entropy. These results have important implications for the interpretation of self-report data in clinical populations. Meta-response markers related to response patterns, rather than the values reported on individual items, may be an informative addition to future research and clinical practise.

## Introduction

### Background

Self-report scales are common measurement tools in neuroscience, psychology, and neurology. Likert scales and Visual Analogue Scales provide a seemingly straightforward, inexpensive, and scalable means to assess patient-based experiences and symptoms. However, there are assumptions which must be met if they are to be valid indicators of the underlying constructs they seek to measure. For example, that respondents engage with the content in such a way as to give meaningful responses.

Violations of the necessary assumptions have been reported. For example, content non-responsivity describes the general phenomenon of participants failing to give meaningful responses in self-report measures. This encompasses ‘random’ or ‘careless’ responding with ‘insufficient effort’^1,2^. These latter terms have implicit associations regarding the style or cause of participant responses and may be pejorative. Indeed, people struggle to respond randomly even when instructed to do so^3^ whilst others may respond inappropriately for more complex reasons than simple carelessness. We will therefore use the term content non-responsivity.

Content non-responsivity rates are approximately 8–12% in studies with healthy populations^4^, and are often higher in clinical groups^5^. Content non-responsivity due to careless responding is sometimes considered a methodological confound, skewing research outcomes, and reducing statistical power^6,7^. However, response patterns may also be measures of interest. For example, Bowling et al.^8^ reported associations between response strategies and personality traits, while Conijn et al.^5^ reported that participants with anxiety disorders engaged in greater levels of ‘satisficing’. We propose that the nature of response strategies provides information above and beyond the raw scores on self-report scales, especially in clinical conditions.

The group of syndromes associated with frontotemporal lobar degeneration (FTLD) may be particularly prone to high levels of content non-responsivity due to their cognitive and behavioural profiles. For example, apathy is a core feature of behavioural-variant frontotemporal dementia (bvFTD)^9^ and a supportive criterion for progressive supranuclear palsy (PSP). Apathy may manifest as insufficient effort responding^5,7,10^. It is particularly pertinent given apathy itself is commonly assessed using a self-report rating scale (e.g. Apathy Evaluation Scale^11^), resulting in a reciprocal impact of motivation on the quality of apathy measures. Social cognition is also impaired in bvFTD^12,13^ undermining the ‘social contract’ between researchers and participants to engage with assessments as intended, leading to a lack of social accountability^14^. This problem similarly arises with healthy participants in anonymised, online questionnaires^15^.

In bvFTD and PSP, impulsive^9^, perseverative, and stereotypic behaviours^16^ could result in repetitive or invariant response patterns. These behaviours are common following damage to, or degeneration of, the prefrontal cortex^17,18^, with difficulties in task switching potentially worsening the ability to engage with sequential response items independently. Deficits in semantic memory^19^ and anosognosia^20^ in bvFTD may similarly result in meaningless responses. People may engage response strategies that minimise effort, using repetition or completely invariant responses^21^.

Despite these concerns, self-report scales have often been used to study bvFTD, PSP, and related disorders in research (e.g.^22,23^) and clinical settings. In this study we aimed to quantify response strategies in bvFTD and PSP and explore their relationship to disease severity, using visual analogue scales (VAS) as an exploratory case study.

### Measures of entropy

After reviewing guidelines for the quantification of content non-responsivity^1,24^, we identified simple tools to assess invariant response strategies, internal consistency and entropy^25^.

Shannon entropy is a construct from information theory that can be intuitively considered as the level of randomness in a dataset. The entropy of a response set may also be considered as the number of responses needed to predict the subsequent response. Completely random responses therefore have higher levels of entropy while stereotyped responses generate low entropy data. Commonly used proxies of Shannon entropy are lossless compression algorithms, such as Lempel–Ziv compression which has been used to evaluate entropy for diverse data types including EEG time series^26,27^ and genetic sequencing^28^. Lempel–Ziv compression reduces the size of a dataset through identifying recurring patterns and is therefore well suited to the problem of identifying patterned response strategies.

However, EEG and genetic sequences are considerably longer than VAS response sets. Shannon entropy and Lempel–Ziv compression may not be appropriate for short strings. The alternative Effort-to-Compress (ETC) metric was designed as a more robust measure for short datasets^29^. This uses the Non-Sequential Recursive Pair Substitution compression algorithm^30^, which operates through iteratively identifying the most common pair of symbols and replacing them with a new symbol. In this case, ETC simply describes the number of steps to reduce a data string to a single digit. We selected this technique as co-primary measure of entropy and included alternatives (e.g. Lempel–Ziv compression) in a planned exploratory analysis.

### Hypotheses

We propose that self-report data from people with bvFTD and PSP will not always be meaningful at face value. Response strategies are more likely to feature invariance or distinct patterns, resulting in lower response entropy. These response strategies have predictive capabilities above and beyond the purported measures of the VAS.

We preregistered our analysis of response patterns in patients with bvFTD and PSP (available at https://osf.io/v4b6n). Though separate disorders, there is a phenotypic overlap between, and heterogeneity within, these conditions^31–33^. We therefore adopted a transdiagnostic approach in considering these two disorders associated with the ‘FTLD spectrum’. Specifically, we tested the hypotheses that:

FTLD participant responses have lower entropy than that of healthy controls.
(A) FTLD participant responses have lower internal consistency than that of healthy controls but (B) there is a greater discrepancy in the measures of entropy than internal consistency when comparing FTLD participants and controls.
(A) There is no overall significant relationship between VAS response values and VAS response entropy but (B) disease severity is a moderator of the relationship between VAS response values and VAS entropy.
Loss of entropy predicts (A) cognitive, (B) behavioural, and (C/D) functional measures in patients, above and beyond VAS scores.

## Results

There was no evidence for a difference in mean age (W = 885.5, BF = 0.27, *p* = 0.53) or sex (W = 898.5, BF = 0.32, *p* = 0.35) between patients and healthy controls (see Table 1). Given the sex distribution of our participants we ran sensitivity analyses within men/women sub-groups. There were no changes in the direction of the effects reported below, although some effects did drop below standard evidentiary thresholds due to a drop in precision in the dataset (c.f. power). Of the 81 participants, only 4 were left-handed. This meant that there was insufficient power to explore potential effects of handedness but note that response biases in the direction of the dominant hand would not affect the analysis of sequential response patterns.

**Table 1:** Age, sex, and visual analogue scale (VAS) score for controls and combined patient group.

	Control (n = 39)		Patient (n = 42)	
	Mean	SD	Mean	SD
Age	66.72	4.55	65.07	9.78
Sex (M: F)	25:14	/	31:11	/
Handedness (L: R)	0:39	/	4:38	/
VAS mean	3.2	0.87	4.37	1.43

Cronbach’s alpha was 0.88 across patients and controls meaning the VAS can be said to have good reliability overall^34^. There was strong evidence for a difference between the mean VAS score between groups (W = 381.5, BF = 21.75, *p* < 0.01), with patients having higher scores than controls. This suggests patients tended to answer with more negative valence.

### Hypothesis 1

**Figure 1:**
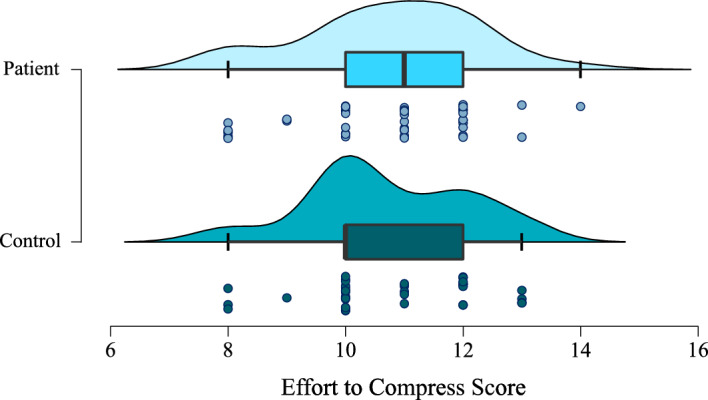
Effort-to-compress scores for patients and controls (BF = 0.23, *p* = 0.61).

There was evidence for no difference in ETC score between patients and controls (W = 791.5, BF = 0.23, *p* = 0.61; see Fig. 1). There was also no evidence in favour of a difference between the two patient groups (W = 232.5, BF = 0.75, *p* = 0.38). However, in the latter case, 0.33 < BF < 3 implying lack of sufficient precision (cf. power) to draw inferences either way.

### Hypothesis 2

**Figure 2:**
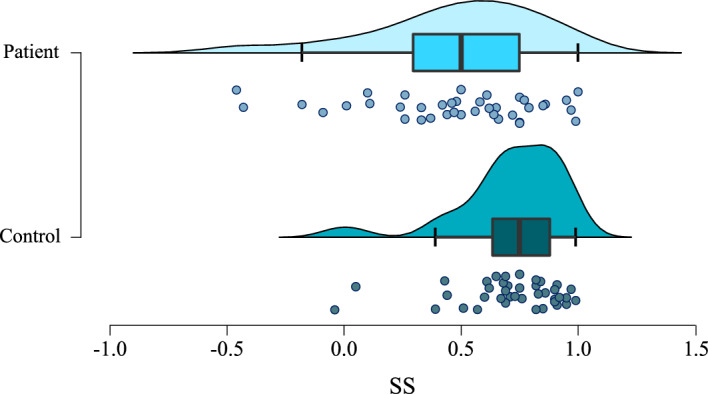
Individual and spread of semantic synonyms scores (SS) for patients and controls (BF = 13.68, *p* < 0.01).

**Figure 3:**
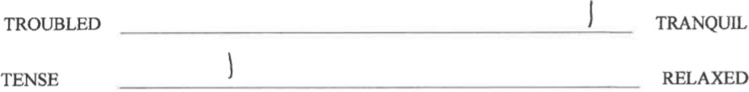
Responses taken from a patient VAS showing internal inconsistency as they mark both very tranquil and tense.

Patients had poorer internal consistency in their responses than controls, with strong evidence in favour of the alternative hypothesis (W = 1080, BF = 13.68, *p* < 0.01; see Fig. 2). An example of this internal inconsistency is evident in Fig. 3, with a bvFTD patient noting they are both very tranquil and moderately tense. There was therefore a greater discrepancy in the measures of internal consistency than entropy when comparing FTLD patients and controls, contradicting hypothesis 2B.

### Hypothesis 3

**Figure 4:**
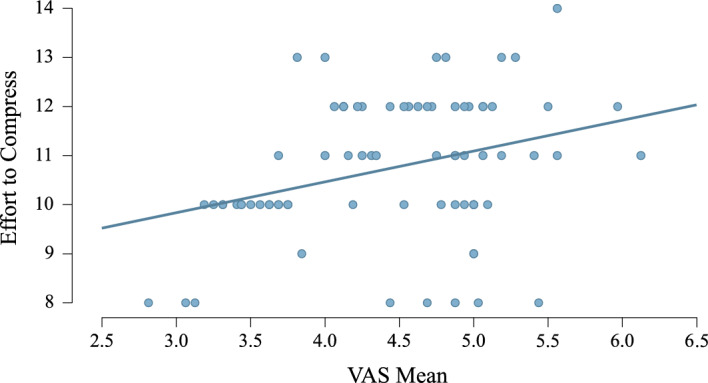
Correlation between effort-to-compress score and visual analogue scale (VAS) mean (r = 0.33, BF = 8.77, *p* < 0.01).

There was very strong evidence for a correlation between VAS mean and ETC score (r = 0.45, BF > 100, *p* < 0.01; see Fig. 4). This correlation was not observed in either patient group independently.

### Hypothesis 4

**Figure 5:**
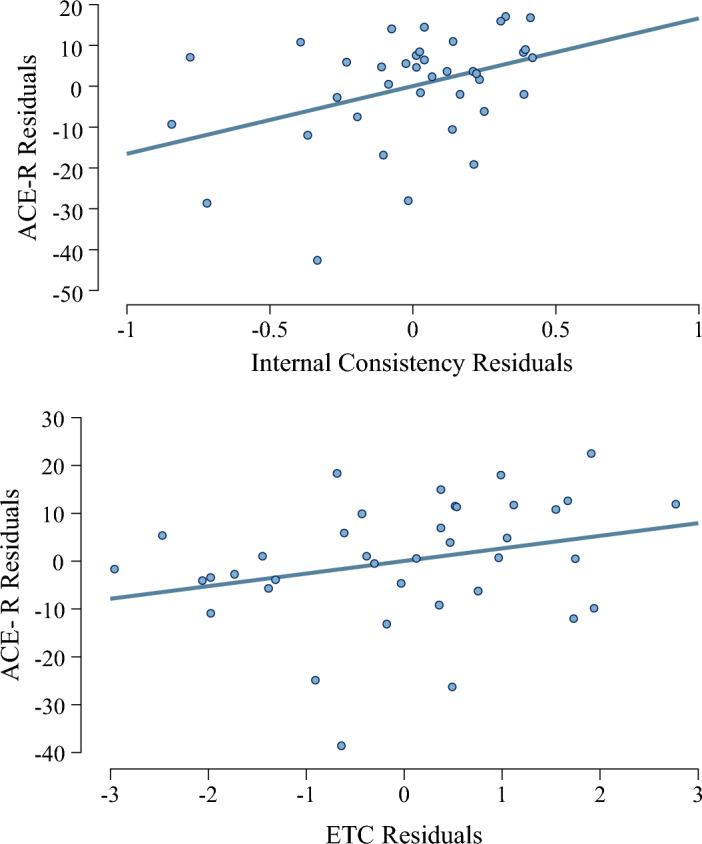
Regression plots showing residuals for the Revised Addenbrookes Cognitive assessment (ACE-R) against residuals for internal consistency and the effort-to-compress algorithm for participants with bvFTD and PSP. Residuals are corrected for age, mean visual analogue score, and invariance score.

In keeping with hypothesis 4A, Bayesian model selection reported moderate evidence in favour of a combined model of semantic synonyms and ETC to be the best predictor of ACE-R score (BF = 5.29; see Fig. 5). Frequentist linear regression using stepwise input corroborated the use of semantic synonyms in an optimal model (t = 2.37, *p* = 0.01), but did not include ETC.

With CBI as the outcome variable for hypothesis 4B, model selection found no combination of input parameters with a Bayes factor above 1.5, meaning there is at best anecdotal evidence for any model to predict behavioural outcomes. In the frequentist linear regression only VAS mean was identified as a significant predictor (t = 3.73, *p* < 0.01).

For predicting function in bvFTD patients using the FRS (hypothesis 4C), there was only weak evidence for the best model which included age, VAS mean, longstring, and semantic synonyms as predictor variables (BF = 2.74). This model is partially supported by linear regression which reported both age (t = 3.68, *p* < 0.01) and longstring (t = −2.30, *p* = 0.04) as significant contributors.

The best model to predict function in PSP patients (PSP rating scale) had only weak evidence, (hypothesis 4D) and featured only longstring (BF = 1.167). This was not corroborated by any frequentist linear regression as none of the variables were significant predictors.

### Exploratory analyses

We repeated our analyses using Lempel–Ziv compression (LZC).

**Figure 6:**
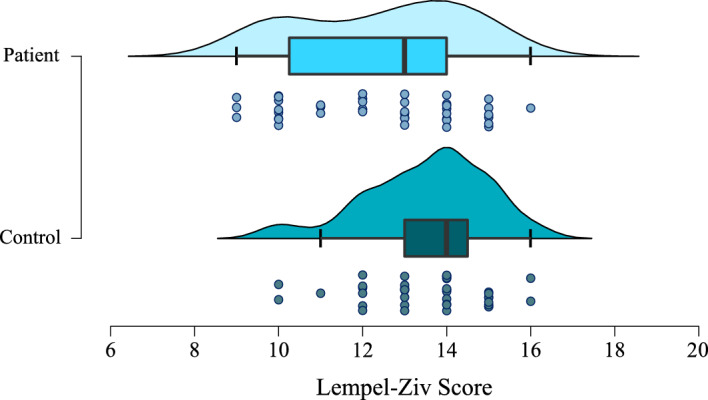
Individual and spread of Lempel–Ziv compression scores for patients and controls (BF = 10.18, *p* < 0.01).

Firstly, there was moderate evidence for a difference in LZC scores between patients and controls (W = 1058, BF = 4.49, *p* = 0.01; see Fig. 6), and moderate evidence that bvFTD patients have lower entropy in their responses than PSP patients (W = 283.5, BF = 4.28, *p* = 0.01). When investigating a correlation between VAS mean and LZC scores, there was no evidence in favour of either hypothesis (r = -0.16, BF = 0.37, *p* = 0.16). Use of LZC score rather than ETC did not make a difference in the optimal model chosen as a predictor of any of the chosen outcome variables.

**Figure 7:**
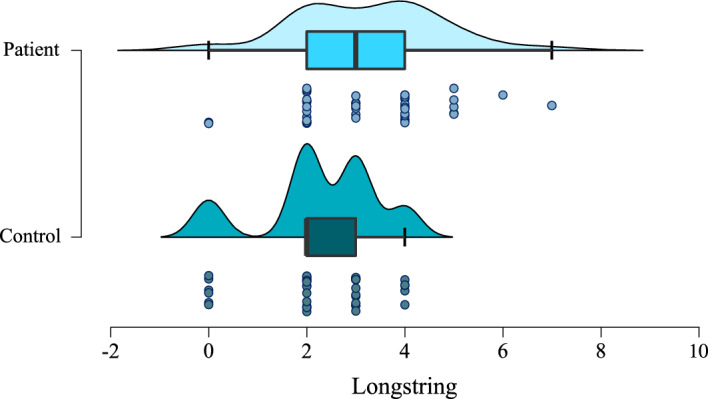
Individual and spread of longstring scores for patients and controls (BF = 15.19, *p* < 0.01).

There was strong evidence of a difference in longstring measures between patients and controls (W = 516.5, BF = 15.19, *p* < 0.01; see Fig. 7).

## Discussion

The principal result of this study is that patients with behavioural-variant frontotemporal dementia and progressive supranuclear palsy differ from healthy controls in the way that they respond on visual analogue scales. There were multiple violations of the core assumption that participants engage meaningfully with self-report metrics. Their *response patterns*, not just the nominal VAS scores, may be indicative of disease and disease severity. Overall, patients used response strategies which were significantly more invariant and less internally consistent than controls. The nature of these response patterns was predictive of cognitive measures in the patient group, suggesting these meta-response markers provide meaningful data over and above the raw measures of the scale.

Internal consistency was poor within our participant responses, for example marking oneself as both tranquil and tense within the same scale. This could be caused by a range of symptoms associated with FTLD, including a lack of insight^12,35,36^, or loss of semantic comprehension^19^. Given that the ACE-R also includes items which test semantic knowledge, internal consistency may predict cognitive scores on this measure through interactions with semantic deficits. Including patients with semantic-variant primary progressive aphasia in future studies would help elucidate the mechanisms underlying inconsistent responding strategies. In particular, contrasting these patients with their behavioural variant counterparts could help distinguish deficits of motivation and insight from impaired semantics.

Invariant responding was a common strategy among patients, which may be exacerbated by motivational deficits. Apathy is a hallmark symptom of bvFTD^18^, and is common across the FTLD spectrum^33^. Given that repeated responses occur faster than alternating responses^37,38^, an invariant strategy may be used to minimise effort, ignoring item content to avoid more effortful task shifting. Invariance may also partially predict scores on the FRS due to both measures targeting participant engagement. For example, one item on the FRS asks whether patients lack interest in daily life. Motor deficits may similarly necessitate an invariant responding strategy as this minimises movement needed to complete the VAS successfully. It would be interesting to explore whether this issue is robust to different self-report modalities and could be overcome by having patients answer verbally to avoid limb-motor deficits.

There was significantly more discord in our measures of patterned responding, with one entropy proxy finding a significant difference between patients and controls, whereas the other did not. After investigating scores for individual participants, ETC seems to be somewhat limited by its algorithmic reliance on pairing items, making it less forgiving to changes in patterns or variance within a recurring motif. For example, whilst Lempel–Ziv compression ratios are the same for ‘1 10 1 10’ and ‘1 10 10 1’, these sequences receive different scores from the ETC algorithm.

Lempel–Ziv compression also takes into account conditional dependencies between items in the scale, whereas ETC processes the data as a whole. Patterned responding in patients may similarly be the result of conditional dependencies between items as patients have difficulties engaging independently with sequences of similar questions. For example, Brugger et al.^39^ previously reported a correlation between dementia severity and sequential randomness in Alzheimer’s disease, such that patients with more severe dysexecutive deficits struggled to generate sequences of independent numbers. Specifically, they report more stereotyped responses which contain “fewer digit combinations”, results reminiscent of the more patterned, low-entropy responding identified in our FTLD patients. More recent research has confirmed the role of the prefrontal cortex in inhibiting the habitual responses needed to generate random sequences^40,41^. Though our study focussed on VAS measures rather than random responding, it is similarly based on the need to inhibit prepotent responses (e.g. answer in keeping with a pattern or invariantly) and switch between tasks effectively^42^. Both of these cognitive mechanisms are impaired in FTLD^21,43–45^. ETC may therefore fail to find patterned responding in our data due to the parallel nature of its compression.

Our findings suggest that researchers and clinicians should be cautious when interpreting results from VAS measures in clinical populations. Future work should continue to explore the benefits of analysing response strategies, as well as the purported measures of the VAS. Given past research connecting content non-responsivity to personality traits^8^, and anxiety^5^, it is evident that exploring response strategies may be pertinent across a wide range of neurological and psychiatric conditions, as well as in healthy populations.

There are limitations to the current study. Firstly, the lack of a well-supported entropy proxy for short data strings was problematic for the analysis of self-report VAS data. Future studies may continue to explore new methods of quantifying content non-responsivity, with a particular focus on entropy measures as proxies of patterned responding.

Moreover, our study included a moderately sized clinical cohort, which may raise the question of power. Bayesian analyses confirmed that our study was sufficiently powered (in terms of adequate precision) as to support or refute the main null hypotheses with at least moderate evidence. However, there were some metrics for which our data was inconclusive, particularly when splitting patient groups, as in the regression analyses of functionality. A further limitation is that our patient group may not be representative of the full FTLD spectrum in all its progressive stages. For example, the majority of our PSP group had Richardson’s syndrome and all patients were sufficiently functional to take part in the source psychopharmacological study. Moreover, we did not include any patients with primary progressive aphasia variants of frontotemporal dementia. Further research should explore response patterns across a wider range of FTLD conditions and severities, ideally confirming diagnoses with pathology where possible. Expanding this research to encompass other neurodegenerative conditions would be useful, noting similar results in Alzheimer’s disease^39^.

This study was also limited to VAS analysis and replicating the analyses for other self-report scales (e.g. questionnaires, Likert scales) would be useful.

In conclusion, people with behavioural-variant frontotemporal dementia and progressive supranuclear palsy violate key assumptions of self-report measures, which we suggest may affect many other clinical population studies. VAS scales should be used with caution in these clinical groups and future studies may choose to quantify meta-response markers, rather than the standard response values, in predicting diagnosis and clinical outcomes.

## Methods

### Participants

Participants’ data were drawn from the baseline assessment in a psychopharmacological study^46^. The study was exempted by the Medicines and Healthcare products Regulatory Agency (MHRA) from clinical trials status. Participants gave written informed consent, and the study protocol was approved by the Cambridge 2 Research Ethics Committee.

All experiments were performed in accordance with relevant guidelines and regulations of the University of Cambridge and Cambridge University Hospitals NHS Trust (the joint sponsor), as well as in accordance with the Declaration of Helsinki. All participants provided written informed consent, or for patients who lacked mental capacity to participate in research, we followed the UK Health and Social Care legal framework with a personal consultee.

Participants completed a VAS to assess potential changes in mood, arousal, and motivation. We analysed the responses from 86 participants who undertook the VAS, of whom 40 were healthy controls and 46 were patients with a diagnosis of bvFTD or PSP (see Adams et al.^47^ for more details). Patients with PSP predominantly had Richardson’s syndrome although PSP-F was also represented^48^.

Only VAS with greater than 50% of measurable responses were included in analysis. Scales with responses that did not fall on the response line (e.g. circling words) were excluded, but responses along the response line other than the suggested vertical mark were included. This led to the exclusion of a control and four patients, leaving 39 healthy controls and 42 patients in the final analysis.

### Materials

**Figure 8:**
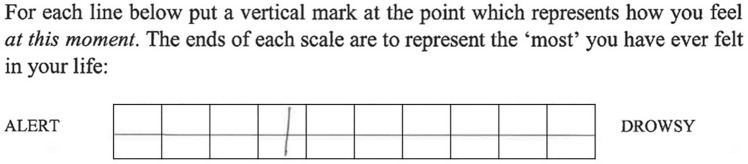
Example extract from the visual analogue scale (VAS). The ‘box’ was not present when completed by participants but was placed for quantification of participant response. In this example the VAS response = 4.

The VAS scale consisted of 16 word pairs (see full list in Table 2). VAS scores were quantified by dividing each response line length into ten equal parts, labelled 1 through 10 (see Fig. 8). The recorded response was the section in which the participant mark crossed the response line. If the response did not cross the line, the recorded response was the point closest to the line. If the participant marked with a cross, the centre of the cross was the recorded response. If the participant mark sat on the boundary between two response categories, the recorded response was an average of those response categories (e.g. between 5 and 6 = 5.5).

**Table 2:** Word pairs featured on visual analogue scale.

Alert	*Drowsy*
*Calm*	Excited
Strong	*Feeble*
*Muzzy*	Clear-headed
Well-coordinated	*Clumsy*
*Lethargic*	Energetic
Contented	*Discontented*
*Troubled*	Tranquil
*Mentally Slow*	Quick-witted
Tense	*Relaxed*
*Incompetent*	Proficient
Happy	*Sad*
*Antagonistic*	Amicable
Interested	*Bored*
*Withdrawn*	Gregarious
Attentive	*Dreamy*

The standard VAS score for each scale was calculated as the mean of participant responses, adjusted for the direction of the word pair valence such that the negative-to-positive direction of effect is aligned (as per italics in Table 1).

### Experimental measures

All analyses were conducted in MATLAB (2021a) using a ‘datascreen’ function that is publicly available on the Open Science Framework (https://osf.io/v4b6n).

Maximum longstring analysis was selected as the measure of invariance and is one of the simplest methods of assessing content non-responsivity by identifying the longest string of repeated responses for each participant.

ETC^49,50^ used the Non-Sequential Recursive Pair Substitution algorithm^30^ as a proxy of entropy, or complexity. We selected this measure as it correlates with the widely used Lempel–Ziv compression algorithm^29,51^, while being more robust to short and noisy sequences^29^.

Semantic synonyms^52^ measure internal consistency by separating VAS measures into pairs a priori, based on semantic similarity. To pair the VAS responses into semantic synonyms, three independent raters separated the VAS items into semantically similar groups. We then analysed the frequency of item grouping across raters, with items most frequently categorized in the same semantic group being established as semantic synonyms (see Table 3). We acknowledge that not all synonyms have perfect mappings, but excluded questions for which a consensus between raters was not reached. We then calculated the correlation between pairs of semantic synonyms for each participant to generate a score of internal consistency.

**Table 3:** Pairs of items used for semantic synonyms.

Pair 1	Pair 2
Alert-Drowsy	Attentive-Dreamy
Calm-Excited	Lethargic-Energetic
Strong-Feeble	Well-Coordinated-Clumsy
Muzzy-Clear Headed	Mentally Slow-Quick Witted
Contented-Discontented	Happy-Sad
Troubled-Tranquil	Tense-Relaxed
Antagonistic-Amicable	Withdrawn-Gregarious

Note that two items are missing from this table as consensus on suitable pairings could not be reached.

We considered the use of other metrics^1,24^. In some cases their key assumptions were violated in our dataset. For example, (I) Mahalanobis D outlier analysis^53^ relies on aberrant responders being identifiable as an exception to the population norm, but we assume that content non-responsivity may be approximately normally distributed in our clinical sample. (II) The odd–even consistency index^54^ relies on correlations between questionnaire subscales, which were not explicitly present in our VAS. (III) Catch trials, and other ad hoc techniques^1^, were not applicable to our dataset. We also considered the use of more traditional self-report evaluation tools (e.g. Cronbach’s alpha) but these methods are designed to assess the validity of the scale itself, and not responses to the scale which was the primary concern of this study. We therefore report Cronbach’s alpha for the VAS but focus our analysis on established measures of content non-responsivity as seen in Curran et al.^1^.

### Analysis

All Bayesian analyses were conducted in JASP version 0.16.0.0 (JASP Team, 2021), with secondary frequentist statistics. We used the default prior option in JASP, with Cauchy distribution and spread r set to 1/√2. Where necessary, assumptions of parametric statistics were evaluated using Shapiro-Wilks test of equal variance^55^ and Levene’s test of homogeneity of variance^56^. A Mann–Whitney test was used if either assumption was violated.

The Bayes Factor (BF) was interpreted in keeping with established conventions^57,58^. Standard evidentiary thresholds were used for moderate (> 3), strong (> 10) or very strong (> 100)^59^ evidence in favour of the alternate hypothesis, or 1/3, 1/10 and 1/100 for corresponding strength of evidence for the null hypothesis.

Hypotheses 1A and 2A were assessed using two Bayesian Mann–Whitney tests, comparing ETC and semantic synonyms respectively between patients and healthy controls. Pseudo *t*-tests were not used as assumptions of equal variance and/or normality were violated in all cases. As we specified a directional hypothesis, we truncated the prior distribution at zero, meaning only positive effect sizes were tested.

We tested hypothesis 3A and 3B by conducting a simple correlational analysis between VAS response mean and ETC score. Finally, we explored hypotheses 4A-D using linear regression with five independent variables in each model: maximum longstring, ETC, semantic synonyms, VAS mean score, and age^60^. The response variables in these three analyses were (4A) the Revised Addenbrookes Cognitive Examination^61^ (ACE-R) to reflect the cognitive domain and (4B) Cambridge Behavioural Inventory^62^ (CBI) to reflect the behavioural domain. Functionality was further divided into two separate analyses due to different validated functional measures being applicable across conditions. Functionality was therefore assessed for 4C using the Frontotemporal Rating Scale^63^ (FRS) for bvFTD, and in 4D the PSP rating scale^64^ (PSPRS) for PSP.

## Data Availability

The analysis code and raw data are available on the Open Science Framework, along with the study preregistration, at the following address https://osf.io/v4b6n. For any further data requests please contact the corresponding author, Rebecca Williams (rsw53@cam.ac.uk).
